# Supplementary material for: Effects of Antiretroviral Treatment on Central and Peripheral Immune Response in Mice with EcoHIV Infection
Source: Cells. 2024 May 20;13(10):882. doi: 10.3390/cells13100882 (PMC11120433; doi:10.3390/cells13100882)
Supplement: Supplementary file 1 [file cells-13-00882-s001.zip › cells-2986554-supplementary.pdf]

Supplemental Figures

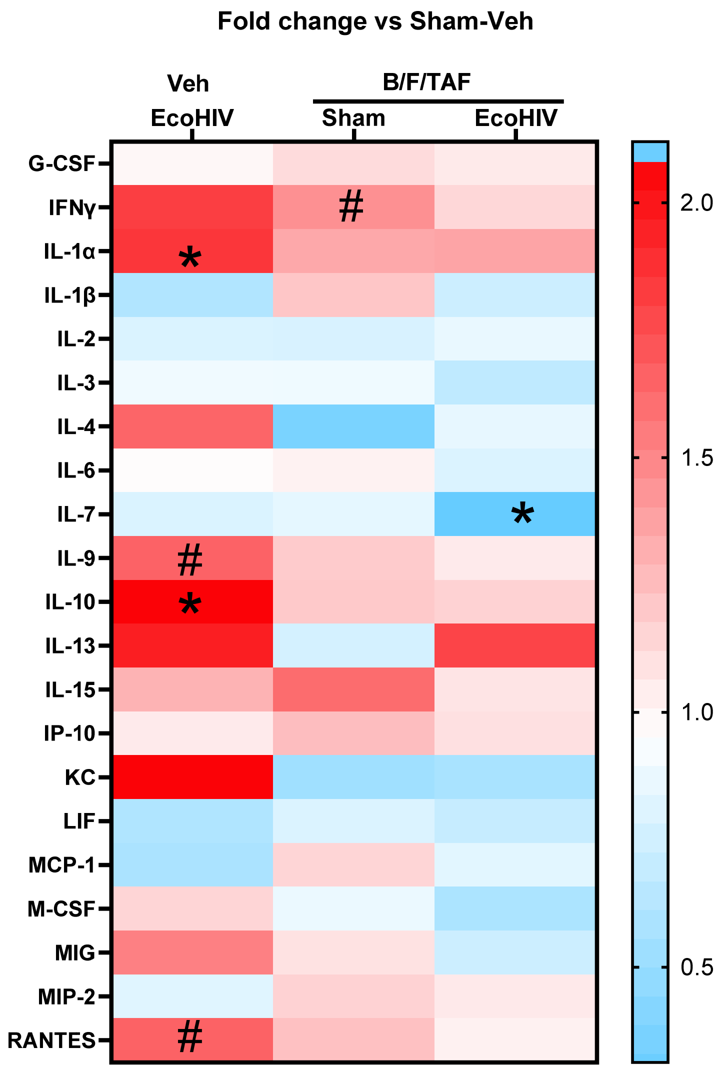

Supplemental Figure S1. NAc expression of inflammatory factors versus vehicle control. Expression of NAc chemokine and cytokines in EcoHIV-Veh, sham-B/F/TAF and EcoHIV-B/F/TAF treated mice represented as the fold-change verse sham-Veh group shown as heatmap (fold increase indicated in red, decrease in blue). n = 5-6/group, *p < 0.05, # p<0.1

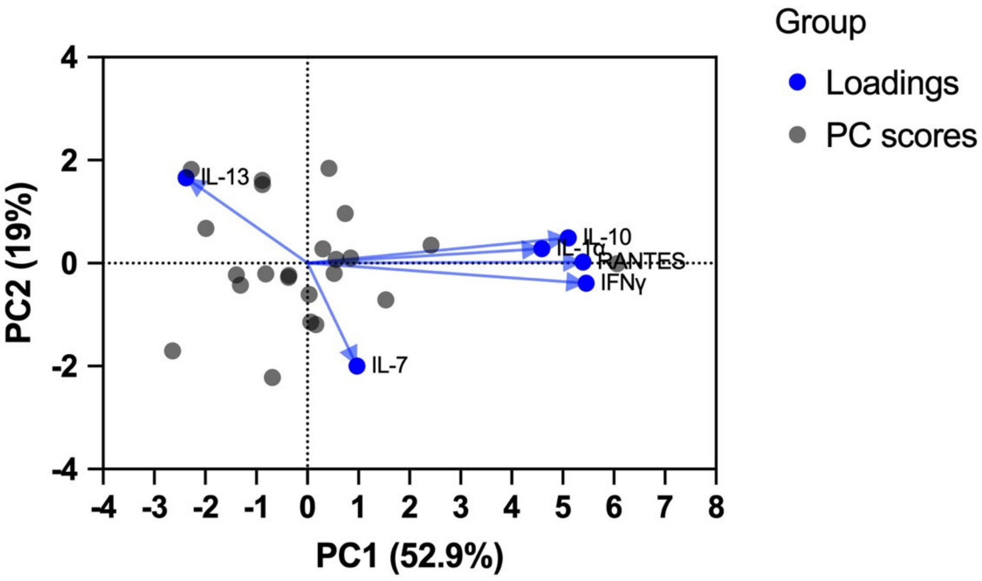

Supplemental Figure S2. Biplot of the attributes of PCA. The biplot shows the impact of each biomarker on each of PCs. IL-1α, IFNɣ, IL-10 and RANTES contribute to PC1 loading and are positively correlated (clustered). IL-13 and IL-7 contribute to PC2 loading and they are negatively correlated (opposite sides of the biplot origin).

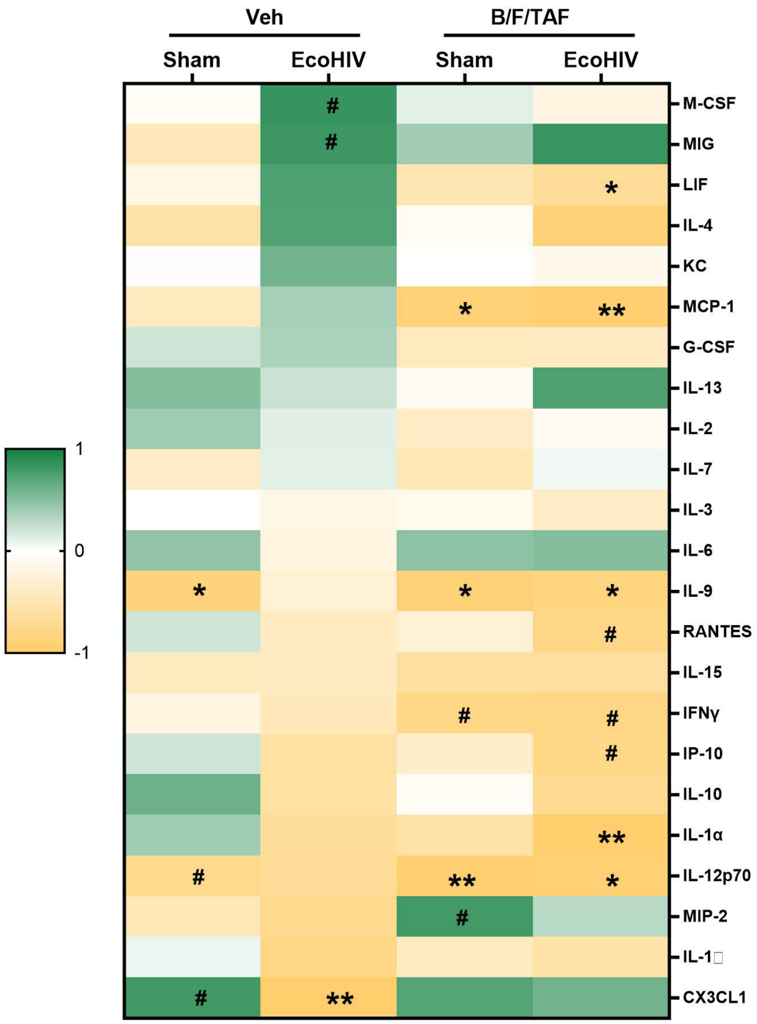

Supplemental Figure S3. The relationship between Iba-1 expression and expression of immune factors in the NAc was modulated by EcoHIV infection and B/F/TAF-treatment status, with distinct patterns of relationships observed in particular in the EcoHIV-infected, vehicle-treated mice. Colors represent Pearson's r values of correlations (green is positive, yellow is negative). n = 5-6/group, *$p < 0.05$, **$p < 0.01$, # $p < 0.1$.

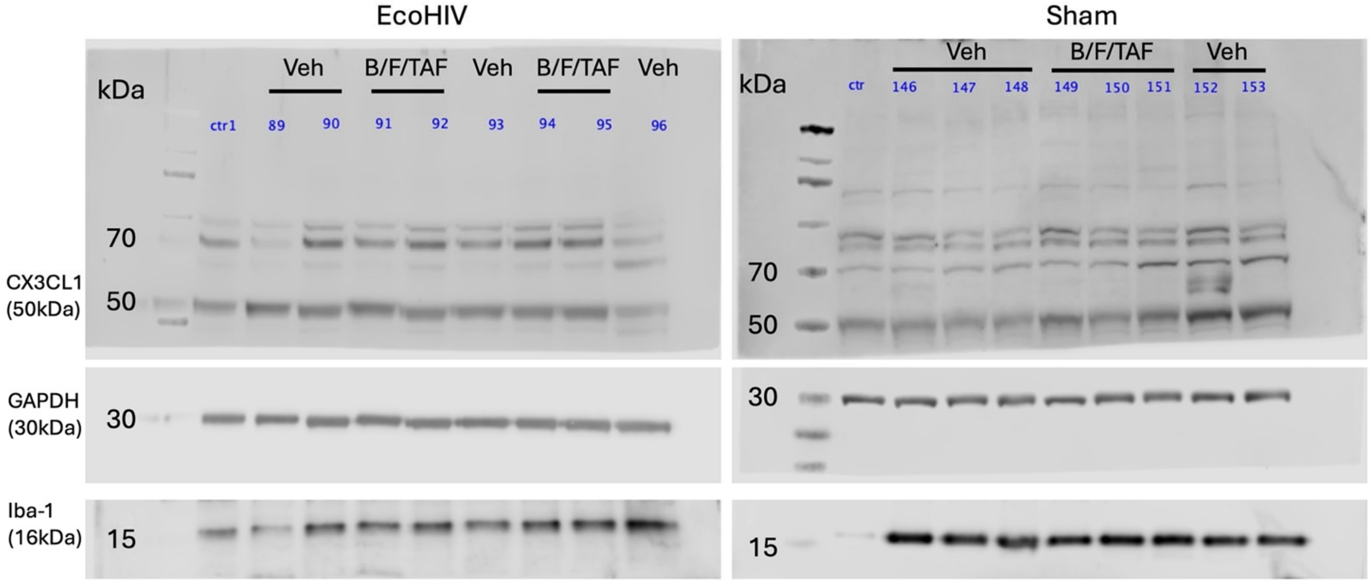

Supplemental Figure S4. The complete western blot for CX3CL1, Iba-1 and GAPDH.

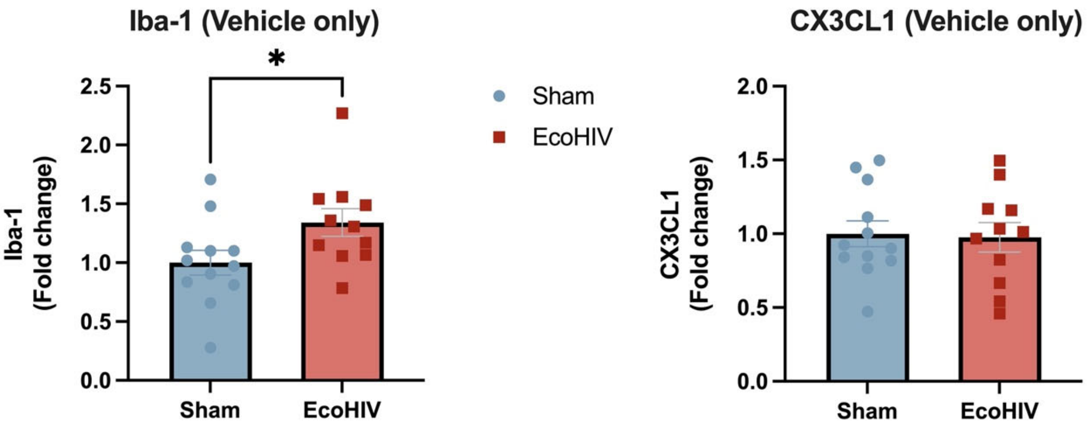

Supplemental Figure S5. Based on an *a priori* hypotheses, overall mean NAc expression of Iba-1 and CX3CL1 were compared between vehicle treated Sham and EcoHIV-infected mice. EcoHIV infected mice exhibited higher levels of Iba-1, but not CX3CL1, n = 11-12/group, $*p < 0.05$.

	Veh	B/F/TAF	
Cytokine	EcoHIV	Sham	Sham
G-CSF	0.77	0.408	0.5471
**IFNγ**	0.1397	#0.0836	0.5793
**IL-1α**	***0.0327**	0.1707	0.1979
IL-1β	0.3444	0.4812	0.4716
IL-2	0.6588	0.35	0.5875
IL-3	0.7068	0.625	0.1123
IL-4	0.7058	0.1122	0.7673
IL-6	0.9347	0.6584	0.1516
**IL-7**	0.6249	0.7159	***0.0442**
IL-9	0.1737	0.6171	0.835
**IL-10**	***0.0259**	0.1766	0.4579
IL-13	0.2682	0.7645	0.3608
IL-12p70	0.3225	0.1601	0.2828
IL-15	0.4335	0.1586	0.732
IP-10	0.4335	0.1586	0.732
KC	0.1904	0.3784	0.4397
LIF	0.2228	0.5405	0.377
MCP-1	0.2785	0.6039	0.6914
M-CSF	0.7513	0.8052	0.3429
MIG	0.4529	0.6741	0.4108
MIP-2	0.4657	0.2078	0.6656
**RANTES**	0.051	0.1698	0.8078

Supplemental Table S1. The value for each analyte (uncorrected) were compared versus the parent control group (sham infection, vehicle treated) by unpaired t-test, the exact p values are presented. n = 5-6/group, *p < 0.05

	Pearson's r	p value
G-CSF	0.002326	0.9916
IFNγ	0.1542	0.5044
IL-1α	0.04578	0.8357
IL-3	0.1439	0.5124
**IL-4**	0.6274	***0.0031**
IL-6	-0.2328	0.285
IL-7	0.07909	0.7629
IL-9	0.01311	0.9526
IL-10	-0.3597	0.0918
IL-13	-0.1339	0.5424
IL-15	0.2839	0.2005
IP-10	0.2657	0.2205
KC	0.1084	0.6226
MCP-1	-0.05824	0.802
M-CSF	-0.1442	0.5115
MIG	-0.08348	0.7049
**MIP-2**	-0.6368	***0.026**
RANTES	-0.1892	0.3992

Supplemental Table S2. The correlation between NAc and plasma level for each immune factor. The Pearson correlation coefficient r and p value are presented. n = 5-6/group, *$p < 0.05$.
